# Effect of low dose nebulized morphine on respiratory function improvement in mustard gas-exposed patients: A double-blind crossover clinical trial study

**DOI:** 10.22088/cjim.13.3.575

**Published:** 2022

**Authors:** Samad Ghodrati, Mohammad Memarian, Shohreh Alian Samakkhah, Masoud Asadi-Khiavi, Aiyoub Pezeshgi

**Affiliations:** 1 Department of Internal Medicine, School of Medicine, Zanjan University of Medical Sciences, Zanjan, Iran; 2 Department of Food Hygiene and Quality Control, Faculty of Veterinary Medicine, Amol University of Special Modern Technologies, Amol, Iran; 3 Zanjan Applied Pharmacology Research Center, Zanjan University of Medical Sciences, Zanjan, Iran; 4 Department of Pharmacotherapy, School of Pharmacy, Zanjan University of Medical Sciences, Zanjan, Iran; 5 Zanjan Metabolic Disease Research Center, Zanjan University of Medical Sciences, Zanjan, Iran

**Keywords:** Dyspnea, Mustard gas, Morphine, Quality of life

## Abstract

**Background:**

Mustard is one of the most destructive chemical gases used in chemical warfare. Several studies showed effectiveness of inhaled morphine as a secondary treatment for the improvement of dyspnea. Therefore, this study aimed at determining the efficacy of low dose inhaled morphine for respiratory function improvement in patients who were exposed to the mustard gas.

**Methods:**

This study was designed as a cross-over double-blinded clinical trial. Patients exposed to mustard gas were randomly assigned into two groups: 1) received 0.4 mg of morphine by inhalation and 2) received 5 ml of normal saline serum as a placebo in the same manner. After a washout period of one week, the first group received the placebo and the second group received morphine for 5 days. Spirometric indices, expiratory flow peak, exercise test, severity of dyspnea, and quality of life were evaluated as respiratory function parameters. Data analysis was done using SPSS software Version 16.

**Results:**

The mean maximum expiratory flow was significantly higher among cases who used morphine in comparison with the placebo group (p<0.05). Moreover, the severity of dyspnea, quality of life, and the frequency of coughing during the day were significantly improved among the recipients of morphine (p<0.05) while the spirometric indices and exercise tolerance tests were similar between the two groups (p>0.05), but the mean peak expiratory flow (PEFR) was significantly higher among the patients receiving morphine than the placebo patients (p<0.001).

**Conclusion:**

The use of inhaled morphine had a significant positive effect on the respiratory system of people exposed to mustard gas. We can use low doses of inhaled morphine to improve the respiratory function of these patients as a secondary therapy.

The most commonly used chemical warfare is Sulfur Mustard (SM) gas (1). In the Iran-Iraq war (1980-1988), Iraqi military forces widely used mustard gas bombardment, and more than 100,000 Iranians were exposed to this gas (2, 3). At present, 34000 Iranians suffer from long-term complications of mustard gas including skin, respiratory, cardiovascular, neovascular, ocular, gastrointestinal, hematologic, immunological, teratogenic and carcinogenic effects (1, 4). Mustard gas is a strong alkalizing agent which destroys DNA non-specifically. Long-term exposure to mustard gas leads to chronic obstructive pulmonary disease (COPD), bronchiectasis, interstitial lung disease, fibrosis (4), bronchiolitis obliterans with organizing pneumonia (BOOP), asthma, large airway congestion and lung cancer other than mesothelioma. Late complications of mustard gas exposure are concentrated mainly on the respiratory tract (1).

Dyspnea reduces the quality and activity in everyday life (5, 6). Morphine is a pure agonist for the opioid receptor (the main analgesic / painkiller related receptor) (7). In most studies, inhaling morphine has been effective as an adjunct treatment for the improvement of respiratory tract infection. Opioid peptides, opioid precursors and their receptors, as found in the CNS, are also present in the lung tissue (8). The inhaling opioids mechanism of action in pulmonary patients may be multifactorial (5, 6). They include anti-anxiety effects (9, 10), pain relief, prevention of ACH release, as well as prevention of bronchoconstriction and secretion of mucus (9), peripheral vasodilatation, reduction of vascular resistance, prevention of response to receptors, reduction of brain stem response to CO_2_ (the primary mechanism of respiratory depression that is induced by opioids) and decreased vasoconstriction reflex due to increased CO_2_ levels (which can reduce dyspnea) (11). The tissue destruction occurring in pulmonary diseases results in the migration of immune cells released by the endogenous opioid ligands activating the opioid receptors in the sensory peripheral terminals that were previously extinguished (12). One of the benefits of administering inhaled morphine is anti-inflammatory effects of agonists in inflammatory conditions (13). Improvement in dyspnea and coughing, increased exercise tolerance, and improving patient quality of life were the results of inhaled morphine use. The benefits of opioids oral inhalation are that they are directly injected into the airways and prevent the hepatic first pass effect metabolism and the side effects (nausea, vomiting, dizziness, constipation) are reduced (11). The disadvantages of inhaled opioids are high inhalation cost, higher complexity, non-portable system due to the need for electricity and lack of proper use of nebulizer (improper breathing unawareness of the patient and companions) (11). 

Unfortunately, there has been no definitive treatment for late complications of mustard gas due to the lack of pathogenesis mechanism of these pulmonary lesions, and in most cases, late complications of mustard gas are symptomatic and opioid administration is one of the therapeutics that control the symptoms. Given the controversy of studies on the use of inhaled morphine on improving respiratory status, the gap of a comprehensive study on the use of inhaled morphine should be filled considering due to the presence of patients’ late complications of mustard gas exposure who are exposed to mustard gas and their illness problems at the later stages. The aim of this study was to determine the efficacy of low dose inhaled morphine in improving dyspnea, cough cessation, increasing exercise tolerance, improving quality of life of patients exposed to mustard gas and achieving an optimum dosage as an effective secondary therapy for improving the respiratory status of pulmonary patients.

## Method

This study was designed as a cross-over double-blinded clinical trial study. The participants were pulmonary patients exposed to mustard gas referred to pulmonologist and lung clinics at the city of Zanjan in 2013. Written consent was obtained from all patients, and they were allowed to leave the study at any time. The Ethics Committee of Zanjan University of Medical Sciences approved the project (ethical code: 10/90-203-01). Drugs sensitivity, use of medications that interferes with the drug used in the study, cardiovascular, renal, hepatic, endocrine and neurological diseases, as well as participation in a simultaneous clinical trial were the exclusion criteria. The participants were selected among the patients with mustard gas induced pulmonary disease (including COPD, bronchiectasis, ILD, fibrosis, BOOP and asthma) referring to pulmonologist and lung clinics. All medical history about taking any drug, addiction or simultaneous illness were asked along with physical examination. 

The required sample size was obtained using statistical formula according to the mean peak expiratory flowrate (PEFR) in the mustard gas exposed group who were placed in inhaled morphine and placebo groups. The variance of each group was estimated to be 0.15 with 95% confidence level and 80% test power was 9 in each group. Considering the low sample size in this study, a cross-over clinical trial was used. Participants were randomly assigned to two groups. The first group received 0.4 mg of inhaled morphine diluted with 4.6 cc distilled water using SHINMED ultrasonic nebulizer (shining world health care co., Taiwan), which was able to produce microaerosol with a diameter of 0.5-6 microns. It was prescribed for 5 days. The second group received 5 ml of placebo (normal saline serum) similar to the treatment group. The patients used similar type of bronchodilator. After a washout period for one week, the first group received placebo and the second group received morphine for the next 5 days. During the study period, the patients were under the supervision of physician and physician-patient relations were blind. To assess the response to treatment, heart rate, respiration, coughing, intensity of dyspnea based on visual analogue scale (VAS) ranging from 0 to 10 (zero for no dyspnea and 10 for severe dyspnea), improvement of quality of life based on horizontal VAS (HVAS) indicating 10 cm horizontal line ranging from zero for low quality of life and 10 for high quality status were measured every day before and after drug administration. Spirometric findings and increased exercise tolerance were measured based on exercise test before and after administration of the drugs. Arterial oxygen content (Bitmos sat801 model, Germany) and PEFR were measured using Breathe-O-Meter (CIPLA, India). These two parameters were measured at 0, 15, 30, 45 and 60 minutes after taking the drug every day during the study period.

Normal distribution of data was first evaluated using the Shapiro-Wilk test. Non-parametric test including Wilcoxon signed-rank test and linear model for Huynh-Feldt test, repeated measures ANOVA were used as well. Results were expressed in terms of mean and standard deviation. The collected data were analyzed by SPSS software Version 16 and in all analyses, the significance level was considered to be less than 5%.

## Results

In this clinical trial, 18 men with pulmonary disease exposed to the mustard gas with mean age of 51.4±10.1 years (range: 42-78 years) were evaluated. The mean body mass index of these patients was 27.9±5.3 kg / m2 (range: 22-33.4 Kg/m2). Table 1 shows the results of exercise test and spirometric indices in the two groups. 

**Table 1:** Comparison of exercise test* and spirometric indices in the follow- up period between the two groups

**Factor**	**Time**	**Group** ^**^		**Mean difference**	**95%CI**	P-value***
		**Morphine**	**Placebo**			
Exercise test	Before intervention	354.4±26.2	355.2±25.9	-0.8	-2.6-1.2	0.43
	After intervention	356.1±27.8	354.6±25.2	1.5	-1.1-4.1	0.24
	P-value	0.28	0.14	-	-	-
FEV1(l)	Before intervention	2.64±0.75	2.63±0.75	0.01	0.02-0	0.11
	After intervention	2.7±0.70	2.60±0.73	0.1	-0.07-0.26	0.22
	P-value	0.59	0.66	-	-	-
FVC (l)	Before intervention	3.76±0.73	3.76±0.72	0	0-0.01	0.17
	After intervention	3.80±-0.74	3.65±0.71	0.15	-0.01-0.33	0.06
	P-value	0.63	0.19	-	-	-
FEV1/FVC (%)	Before intervention	69.2±11.6	69.1±11.6	0	-0.01-0	0.11
	After intervention	70.2±9.5	64.8±18.6	5.4	-3.5-14.2	0.22
	P-value	0.53	0.30	-	-	-
MMEF	Before intervention	1.96±1.09	2.06±1.06	0.1	-0.05-0.27	0.17
	After intervention	2.01±0.92	2.01±1.24	0	-0.33-0.33	0.99
	P-value	0.69	0.64	-	-	-

^*^Data are mean±SD.

^**^Ten persons in each group.

^***^P<0.05 was considered as statistically significant.

Comparison of 6-Minute Walk Test, forced expiratory volume in the beginning seconds (FEV1), Forced vital capacity (FVC), mean of FEV1 / FVC ratio, and maximum expiratory flow (MMEF) in both intervention and placebo groups showed similar results before and after the treatment, and there was no significant difference between them (p>0.05). At the end of the course of consumption, the difference between the mean changes in the distance traveled by 6 minutes in patients receiving morphine and placebo (1.7 m/min increase versus 0.6 m/min) was not statistically significant (P=0.17). The mean of forced expiratory volume in the first second (FEV1) of the patients in the morphine group increased by 0.06 L and the placebo group decreased by 0.03 L, but the difference between the mean changes was not statistically significant (P=0.27). There was no statistically significant difference between the mean of FVC changes in pulmonary patients in the morphine and placebo groups (P=0.06). At the end of treatment period, the FEV1/FVC mean ratio of the patients in the morphine group increased by 1 unit and decreased by 4.4 units in the placebo group, but there was a 5.4-unit difference in the mean of the two groups which was not statistically significant (P=0.21). Also, the difference of 0.1 L/s between the mean changes in maximum expiratory flow (MMEF) of pulmonary patients in the two groups of morphine and placebo was not statistically significant (P=0.57). Table 2 shows the information on vital signs, frequency of coughing, severity of dyspnea and the quality of life of patients. Based on the results of the Hyun-Feldt test, there was a significant relationship between the use of morphine and the decrease in mean heart rate (HR) and respiratory rate (RR) of patients (p<0.001), but there was a no significant difference between mean heart rate (HR) and the respiratory rate (RR) of patients receiving morphine and placebo (p>0.05). Following the use of morphine, the mean number of coughs a day of pulmonary patients decreased (p<0.001). Also, the mean number of coughs per day in patients receiving morphine was significantly lower than those receiving placebo (P=0.004). Comparison of the severity of the patients' dyspnea using the Visual Analog Scale (VAS) indicated a significant improvement in the respiratory state of the pulmonary patients receiving morphine (p<0.001).

In addition, the severity of the dyspnea of the pulmonary patients receiving placebo was significantly more severe than those receiving morphine (P=0.011). There was a significant relationship between morphine consumption and quality of life of patients (P=0.004). Also, the mean scores of quality of life in patients receiving pulmonary morphine were significantly higher than those receiving placebo (p<0.001). 

Table 3, based on pulse oximeter findings, there was no statistically significant relationship between the amount of time elapsed with morphine consumption and the mean arterial oxygen content during each heart rate (P=0.07). Also, there was no statistically significant difference between the mean arterial oxygen content during each heart rate among the patients receiving morphine and placebo (P=0.73). According to spirometric findings and Hyun-Feldt test results, there was no statistically significant relationship between the time elapsed from taking morphine with mean peak expiratory flow (PEFR) (P=0.32), but the mean peak expiratory flow (PEFR) was significantly higher among patients receiving morphine than that of placebo patients (p<0.001).

**Table 2:** Comparison of heart rate*, respiratory rate, cough, severity of dyspnea, quality of life within and between two groups

***P-value	**Day 5**	**Day 4**	**Day 3**	**Day 2**	**Day 1**	**Before intervention**	**Group** ^**^	**Factor**
0.808	82.6±8.8	85.3±8.6	82.7±8.2	85.6±8.2	82.5±8.6	85.7±8.9	Morphine	Heart rate (frequency per min)
	83.7±9.1	84.8±9.1	84.7±9.1	84.8±9.1	84.6±8.8	84.7±8.8	Placebo	
0.070	15.1±1.3	16.2±1.4	15.2±1.4	16.4±1.6	15.6±1.4	16.7±1.6	Morphine	Respiratory rate (frequency per min)
	16.8±1.7	16.8±1.5	16.8±1.5	16.9±1.6	16.6±1.7	16.7±1.6	Placebo	
0.004	4.0±0.9	4.3±1.2	4.5±0.8	4.8±1.0	5.0±1.0	5.8±1.2	Morphine	Coughing in day
	5.6±0.8	5.4±0.7	5.8±1.2	5.6±0.8	5.5±0.8	5.8±1.2	Placebo	
0.011	4.8±1.2	6.3±0.8	4.34±1.0	5.0±1.0	5.6±0.8	5.6±0.8	Morphine	Severity of dyspnea
	5.6±8.0	5.4±0.7	5.8±1.2	5.6±0.8	5.5±0.8	5.6±0.8	Placebo	
0.001>	7.2±1.2	7.0±1.2	6.7±0.8	6.3±1.1	5.9±1.1	5.6±0.8	Morphine	Quality of Life
	5.7±1.1	5.6±0.8	5.4±0.7	5.8±1.2	5.6±0.8	5.7±1.1	Placebo	

^*^Data are mean±SD.

^**^Ten persons in each group.

^***^P<0.05 was considered as statistically significant.

**Table 3:** Pick flow meter* and pulse oximeter over the time in total 5 days after inhalation in two groups

**Factor**	**Group** ^**^	**0 min**	**15 min**	**30 min**	**45 min**	**60 min**	^***^ **P-value**
Arterial oxygen saturation (%)	Morphine	92.73±1.70	92.71±1.69	92.70±1.67	92.71±1.71	92.69±1.69	0.73
	Placebo	92.64±1.72	92.64±1.71	92.78±1.63	92.89±1.58	92.99±1.57	
Pick expiratory flow rate(Lit/min)	Morphine	264.7±47.90	265.10±47.50	267.20±47.62	268.40±47.80	268.30±46.10	<0.001
	Placebo	234.40±32.10	232.90±33.81	234.10±33.71	234.10±33.22	234.41±33.61	

^*^Data are mean±SD.

^**^Ten persons in each group.

^***^P<0.05 was considered as statistically significant.

## Discussion

Pulmonary complications are the most annoying complications of mustard gas. In several studies, the most common side effects were COPD, bronchiectasis, asthma, major airway stenosis, pulmonary fibrosis and chronic bronchitis (14). Unfortunately, there is no antidote for mustard gas (1). However, there are studies on the benefits of some antioxidant substances in reducing acute pulmonary complications and corticosteroids in chronic bronchitis (8). In our clinical trial, we evaluated the therapeutic effects of low doses of inhaled morphine on respiratory function and the quality of life of chemical veterans exposed to mustard gas. . The mechanism of action of morphine is related to its receptors located in the epithelium of the trachea and large bronchi. Three main opioid receptors have been identified in the respiratory tract: μ (MOR), δ (DOR), and k (KOR), which mediate the effects of the 3 primary families of endogenous opioids (endorphins, enkephalins, and dynorphins, resp.) as well as exogenous opioids such as morphine and codeine. In addition, the lungs also may contain a novel opioid receptor. Among them the k receptor is the predominant opioid receptor in the lung. An additional suggested mechanism for the therapeutic effects of inhaled morphine might be the inhibition of pulmonary-irritant receptors (2).

In our clinical trial, based on peak flowmetric findings, the mean PEFR among patients receiving morphine was significantly higher than the placebo group, but spirometric indices did not show any significant difference between the two groups. In the study of Shohrati et al. (2012), on 40 pulmonary patients exposed to mustard gas, the maximum expiratory flow and spirometric indices in patients receiving inhaled morphine were significantly higher at the end of the study than in the placebo group (15). The results of a number of other studies indicated that morphine has no positive effect on respiratory parameters in patients. In the case of lung functionality, the effect of inhaled morphine in multiple doses (1, 4, 10mg) did not show significantly different between placebo and treatment groups in the BeauFord et al.’s study that was designed as a crossover clinical trial (16). Another study by Harris et al. on 6 ILD patients showed that doses of 2.5 and 5 mg of morphine, compared to placebo, showed no improvements in respiratory function (17).

In Light et al.’s study, oral morphine had no significant effect on pulmonary function compared to the placebo (18). On the contrary, in a series of studies, the beneficial effects of oral and inhaled morphine have been supported. It is noteworthy that even in studies with no favorite conclusion, low doses of inhaled morphine had little side effects. Farn Combe et al. tested different doses of inhaled morphine from 1 mg to 30 mg on 54 cases. In this study, 63% of the patients reported positive results in an increase in respiratory function (19). In our study, the severity of dyspnea based on the scores obtained from the VAS system showed a significant improvement in the respiratory state of the pulmonary patients receiving morphine. Also, in terms of quality of life, there was a significant relationship between drug consumption and patient’s quality of life. Additionally, after using morphine, the cough frequency during the day significantly decreased. In the study of Shohrati et al, the use of inhaled morphine had a significant effect on improving quality of life, reducing the frequency of cough and improving dyspnea (15).

In addition, a meta-analysis (2002) study was conducted on 18 double-blinded clinical trials related to the treatment with opioids which showed that opioids had statistically significant effect on the dyspnea rate reduction. The study also identified more effects for oral and injectable opioids. This study showed that there was not enough data available for the effects of inhaler opioids (20). In another report presented by Cohen et al., morphine was used for dyspnea at doses of 2.5 to 12.5 mg, and moderate improvement was also observed (13). In the studies of Light et al. (18), Zeppetella (21) and Abernethy (8), the severity of dyspnea was reduced among morphine users. However, in contrast to our results, in the study of Masood et al., no significant difference was shown after receiving 10 and 25 mg of inhaled morphine compared to the placebo in the case of dyspnea rate (22). In the double-blinded study of Abernethy (2007) on 48 patients with COPD, patients were treated with 20mg of oral morphine, and there was improvement in the morning and afternoon dyspnea. Most participants reported having improved sleep. The slow-release oral low-dose morphine significantly improved recurrent dyspnea (8). In our study, the results of the 6-minute walking test (exercise test) showed that there was no significant difference between the mean distance traveled in 6 minutes before and after the onset of treatment in the two groups receiving morphine and placebo. Contrary to these results, and Farn Combe et al. showed a significant positive effect of morphine on exercise tolerance test (18, 19). Some of these variables explained the relationships to biologic background of patients and it is clear to consider genetic patterns using molecular biomarkers like gap junction molecule’s role in epithelial cells integrity (23) or bioinformatics approaches in subsequent years (24). 

In this study, it was found that the use of inhaled morphine has a significant positive effect on the maximum expiratory flow rate, severity of dyspnea, coughing and quality of life of patients exposed to mustard gas, while there was no significant effect on spirometric indices and exercise tests. Regarding the results of this study, we can use low doses of inhaled morphine to improve respiratory function in these patients. Of course, it should be considered that taking higher and longer doses will also increase the risk of addiction. It is recommended that studies with higher sample size and longer follow-up should be undertaken to achieve the beneficial effects and possible side effects of morphine inhalation.

## Funding:

This study was a part of internal medicine residency thesis which was financially supported by Department of Internal Medicine, School of Medicine, Zanjan University of Medical Sciences (grant No: 10/90-203-01).

### Conflict of interest:

There was no conflict of interest. 

### Author contribution:

S. Ghodrati, conceived of the study, M. Asadi-Khiavi, initiated the study design and helped with implementation. M. Memarian, data collection and drafting article. Sh. Alian Samakkhah, provided statistical expertise in clinical trial, data analysis and interpretation. A. Pezeshgi critical revision of article and approval of article. All authors contributed to refinement of the study protocol and approved the final manuscript.
